# Does predicted age at peak height velocity explain physical performance in U13–15 basketball female players?

**DOI:** 10.1186/s13102-022-00414-4

**Published:** 2022-02-08

**Authors:** Karol Gryko, Jakub Grzegorz Adamczyk, Anna Kopiczko, Jorge Lorenzo Calvo, Alberto Lorenzo Calvo, Kazimierz Mikołajec

**Affiliations:** 1 Department of Sport Games, Józef Piłsudski University of Physical Education in Warsaw, Marymoncka 34, 00-968 Warsaw, Poland; 2 Department of Theory of Sport, Józef Pilsudski University of Physical Education in Warsaw, Marymoncka 34, 00-968 Warsaw, Poland; 3 Department of Human Biology, Józef Pilsudski University of Physical Education in Warsaw, Marymoncka 34, 00-968 Warsaw, Poland; 4 Department of Sports, Faculty of Physical Activity and Sport, Universidad Politécnica de Madrid, Madrid, Spain; 5 Department of Basketball and Football, Jerzy Kukuczka Academy of Physical Education, Katowice, Poland

**Keywords:** Maturity timing, Adolescent development, Athletes, Physical fitness, Aptitude

## Abstract

**Background:**

The aims of the study were (1) to identify the physical fitness and basic anthropometric characteristics of Polish female basketball players aged 13–15 years, (2) to show the effect of maturity timing on the performance in motor tests and basic body composition parameters, (3) to identify the index that contributes most to the prediction of performance in the tests of speed, jumping ability, agility, and endurance.

**Methods:**

The sample included 904 female Polish players (U13–15). In part 1, maturity timing category distribution were examined within across age-groups. Maturity timing was followed by grouping with respect to years before or after the observed peak high velocity (PHV): PHV0 (− 0.50 to 0.49), PHV1 (0.50 to 1.49), PHV2 (1.50 to 2.49) and PHV3 (2.50 to 3.49). In part 2, the relationship between the anthropometric variables, physical fitness performance was assessed based on maturity timing categories (ANCOVA analysis). In part 3, backward stepwise multiple regression analyse quantified the relationship between maturity timing (group of PHV) and physical performance.

**Results:**

ANCOVA results (age, body height, and body mass as covariates) showed in the U13 female basketball players significantly higher sprinting (20 m), jumping ability and endurance tests results of the PHV1 group. Better results was observed in U14 female players in PHV1 compared to PHV2 and PHV3 in 20 m and jumping tests but opposite trend was observed for 5 m sprint and endurance test (distance covered and VO2_max_). U15 basketball players from the PHV3 group were characterized by better results of jumping abilities, endurance, 10 m and 20 m sprint and agility (total, S_4_) tests. Maturity timing (10 m), chronological age (5 m, 20 m, agility, SVJ, VJ, and VO_2max_ tests), body height (10 m), body mass (10 m, 20 m, VJ, VO_2max_), and the interaction between body mass and height (SVJ) were significant (adjusted *R*^*2*^ = 0.02–0.10; *p* < 0.001) predictors of motor skills.

**Conclusion:**

Trainng content of female basketball players aged 13–15 years old should be adjusted to biological requirements especially in jumping, endurance and 20 m sprint test. The time from peak height velocity (PHV) was a significant predictor only in the 10 m sprint test.

## Background

Basketball is a sport characterized by intermittent high-intensity exercise [1, 2], whereas optimal performance in basketball is achieved through a complex combination of technical and tactical skills and high physical fitness [3]. Athletes who train basketball, the need to analyze many variables such as physical and physiological attributes (body height, body mass, somatotype, body proportions, aerobic profile, strength, anaerobic power, agility, and speed) is emphasized [4]. Talent identification requires multifactorial analyses of several biological [5–7], functional, behavioural and perceptual variables [8] and those related to the training process [9]. Studies indicate the validity and necessity of taking into account chronological age and predicted age at peak height velocity (APHV) of basketball players to optimize the process of identifying gifted individuals [10].

Maturity status (early, on time, late, mature based on skeletal age, stage of puberty) refers to the state of biological maturation of an individual at the time of observation, whereas maturity timing refers to the ages when specific maturational events are attained (ages at peak height velocity and menarche) [11, 12].

Among a large group of those willing to participate in the competitions in team sports, very few athletes reach the highest level of sports skills, while talent identification and qualification for a sport are based on broad criteria selected through scientific analysis [13]. Many studies have emphasized biological variability in sports for young athletes and emphasized it as one of the important aspects of talent identification and sports qualification process [14–17].

In recent years, studies of young basketball players have among others focused on the assessment of trends of anthropometric traits and physical performance [18], longitudinal changes of functional capacities [19], analyses of the effects of different training methods and protocols on power, speed, and anaerobic capacity [20–22]. Interactions between body size and composition in predicting basketball performance have also been considered [23]. These studies are often conducted independently of the maturation rate assessment and the interpretation of the performance of young athletes during adolescence is very important for coaching practice because results can be misinterpreted due to discrepancies between chronological age, biological age, and athletic age resulting from training experience [24].

From a somatic point of view, faster-maturing boys and girls are taller and heavier than their peers of the same chronological age, which gives them a huge advantage in sports involving physical contact. This variation is most noticeable between the ages of 11 and 16. Adolescence is the period in which these differences are more pronounced and the age of 13 to 16 years appears to be the most heterogeneous period [13, 16, 25].

As a factor affecting basketball-specific functional abilities and skills, the interindividual differences in biological maturation have not been regularly evaluated, especially in young female athletes. However, research findings indicate the relative age effect on the success of youth basketball teams [26–28]. Overrepresentation of athletes born in the first months of the year in all age groups has been shown [29]. Relatively older athletes are often characterized by greater body height, which is critical in basketball [30], and an advantage in motor skills, which may bias the assessment of fitness potential of players born in different quarters of the year [31, 32]. The above correlations are most commonly reported in basketball players [30], while this effect has not been sufficiently studied in female basketball players to date. Sexual dimorphism underlies much of the physiological response to physical exercise. Physiological characteristics of girls change with age and puberty due to a different hormonal environment that begins early in fetal life [33]. Participation in an intensive training program for girls during the progressive stages of ontogenesis requires a great deal of knowledge from coaches regarding the functional abilities of young athletes in conjunction with taking care of their general health. In the case of basketball, the empirical findings on these issues are quite scarce [33]. Large differences between girls in terms of the time and pace of biological maturation can create difficulties in the correct interpretation of the rate of acquisition of individual motor and technical skills, and psychological preparation for sports competition [19].

Given the available research findings (very few concerning young female basketball players), there is a strong need for physical fitness testing in basketball on large research samples, especially to identify talents [34, 35]. Therefore, testing was carried out to evaluate the motor potential and basic anthropometric characteristics of a very large population of young female players aged 13–15 years, who were members of Polish basketball clubs. The second study aim was to show the impact of maturity timing on the results of motor tests and basic body composition. The third aim was to identify the index that mostly affects the prediction of performance in the individual tests evaluating speed, jumping ability, agility, and endurance.

## Methods

### Participants

The study sample size was 925 female basketball players aged 13 years (n = 277; age: 13.05 ± 0.28; basketball experience: 3.0 ± 0.8 yrs), 14 years (n = 374; age: 13.95 ± 0.30; basketball experience: 3.6 ± 1.1 yrs), and 15 years (n = 274; age: 14.82 ± 0.28; basketball experience: 4.3 ± 1.3 yrs). All examined athletes belonged to the Caucasian ethnic group. The players were female members of 49 sports clubs competing in the national championship in the age categories U13 and U15. The U14 girls also participated in the national championships at the club and regional competition levels. This group included female basketball players who were members of the national team in their age categories (U13, U14, U15). All girls at this training stage (club training programs) were characterized by a similar training volume (8 h 45 min per week—3 technical training sessions, 1.5 h each, 3 strength and conditioning sessions, 45 min each, and 2 h a week playing games) over a 8-month season (October to May). The examinations were carried out in 2017–2020 during in regular season in the same periods of the year (from November to February) to complete the measurements before the play-off phase.

### Procedure

All of the participants, legal guardians, clubs and Polish Basketball Association were informed in writing about the aims, benefits, and procedure of the research project, and about the possibility to withdraw from the study at any moment. The inclusion criterion was the written informed consent obtained from each participant, and the exclusion criteria were contraindications for the basic anthropometric measurements. Injuries or trauma were also the exclusion criteria. The research was approved by the local Ethics Committee for Scientific Research (SKE 01-28/2016), and the study was conducted according to the rules and regulations of the Declaration of Helsinki [36].

### Biological maturation

The APHV of the female basketball players analyzed in the study was estimated by subtracting the maturity offset from chronological age at the time of measurement [37]. The predicted maturity offset (years) was calculated as $-7.709133 +\left(0.0042232\mathrm{ x }[\mathrm{age}*\mathrm{stature}]\right)$, with standard errors of the equations of 0.542 years [38]. This equation was derived after calibrating the original equation proposed by Mirwald et al. [37]. Early maturers, average maturers, and late maturers were defined as players with an estimated APHV of less than 13.1 years, 13.1–15.1 years, and more than 15.1 years, respectively [39]. Since the average maturers accounted for 97.7% of the basketball players, it was decided to analyze only this group. Due to very low numbers, eight early maturing U13 players, four U14 players (3 early maturers, 1 late maturer), and seven U15 players (6 early maturers, 1 late maturer) were excluded from the analysis. This was followed by grouping with respect to years before/after the observed PHV0 (− 0.50 to 0.49), PHV1 (0.50 to 1.49), PHV2 (1.50 to 2.49), PHV3 (2.50 to 3.49) [39]. After this stage, the decision was made to exclude other two U13 female players from the analysis, who were the only female basketball players in the PHV0 group. Finally, 904 basketball players were considered for comparison.

### Measurements

Body height (cm) was measured barefoot with the head positioned to the Frankfurt plane, using a stadiometer (Seca 264, Seca GmbH & co. kg, Germany) with a precision of 0.1 cm, as were standing reach measurements (Seca 216, Seca GmbH & co. kg, Germany). Body mass was measured using a JAWON Medical X-Scan Plus II device (Certificate No. EC0197 for medical devices) with a precision of 0.1 kg. The measurements were taken by an anthropometry expert who holds an ISAK Level 1 accreditation according to the standards proposed by the International Society for the Advancement of Kinanthropometry (ISAK) [40]. The basic anthropometric measurements and warm-up were followed by physical fitness tests performed each time in the same order (speed test, jumping ability test, agility test, endurance test). Fitness was evaluated in two stages: the morning session (to measure speed, jumping ability, and agility), and the evening session (measurements of endurance) so that adequate rest periods were maintained.

### Speed

Speed was measured using a 20 m sprint test with a split time recorded at 5 m (starting speed) and 10 m, when players ran at full speed. Each participant performed two trials, with the best used as a test result [41]. The photoelectric cells Fusion Smart Speed System (Fusion Sport, Coopers Plains, QLD, Australia) were used to record time (s). The photocells were installed at the starting line, 5 m, 10 m, and 20 m. The time measurement was performed with an accuracy of 0.001 s. Running started from the standing position, with the preferred foot positioned in front. No bouncing and backward movements were allowed before the sprint.

### Jumping

The results of both standing jump (SVJ) and the a vertical jump (VJ) were measured using a yardstick vertical jump device [42–45]. The device is used to measure the height to which a player can push away small sticks placed horizontally on a pole during a jump. Reaching height was subtracted from the height reached while jumping. The first step was to perform 2 standing jump tests. Next, the player had 6 attempts (2 jumps with the dominant leg, 2 with the non-dominant leg, and 2 with both legs), with sufficient rests between jumps. The highest attempt scores were retained for analysis. This VJ protocol has established reliability [42–45].

### Agility

**Fig. 1:**
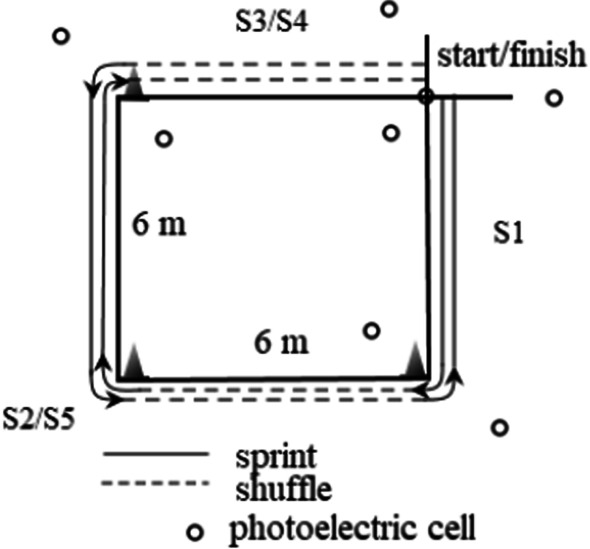
Agility test design

The design of the agility test is presented in Fig. 1. This is a modified Lane Agility Drill [46] test, with the length and width changed to 6 × 6 m to ensure that the proportions between defensive shuffle and sprint were identical. The position of the photocell at the change of direction line was 1 m from the line. The test was repeated twice, and a 10-min rest break was administered to minimize fatigue. Before the test, the participant was familiarized with the procedures by performing a trial (pre-test). The best time achieved during the test was recorded for the analysis of the results.

### Endurance

The Yo-Yo Intermittent Recovery Test level 1 (Yo-Yo IR1) was used to evaluate endurance using a protocol presented in the literature [47, 48]. The total distance covered (m) during Yo-Yo IR1 was the main measure of results and the maximum oxygen uptake (VO_2_max) calculated according to the formula: VO_2_max = IR distance (m) × 0.0084 + 36.4 [48].

### Statistical analysis

The normality of distribution was verified by the Shapiro–Wilk test, whereas the assumption of the equality of variance was verified using the Levene test. The reliability coefficient for the measurements was *Cronbach’s α* = 0.91. The analysis of variance (ANOVA) was employed to show significant differences between the groups of female basketball players. Furthermore, ANCOVA analysis was used to demonstrate the differences in years after observed APHV, with chronological age, height, and mass used as covariates. Bonferroni adjustments were made for post-hoc comparisons. The effect size was evaluated using partial eta squared (η^2^) and classified as: no effect = 0 to 0.039, minimum effect = 0.04 to 0.24, moderate effect = 0.25 to 0.63, and strong effect =  ≥ 0.64 [49].

Backward stepwise multiple regression was used to estimate the relative contributions of chronological age, maturity timing (stage of APHV), body height, body mass, and height x mass interaction (based on residuals) to the variability of individual physical fitness tests. The significance of the effects was set at *p* < 0.05 for all the analyses. All calculations were performed using STATISTICA software (v.13.3, StatSoft, USA).

## Results

When analyzing the variation of female basketball players with respect to age (Table 1) in the U13 group compared to U14 and U15, there were significantly (*p* < 0.001, minimum effect) lower values of age at PHV (by 0.13/yrs and 0.38/yrs, respectively; F_(2,901)_ = 72.1; η^2^ = 0.14), body mass (by 5.5% and 7.7%, respectively; F_(2,901)_ = 21.2; η^2^ = 0.05). In the same comparison there were also significantly (*p* < 0.001, but no effect) lower values of body height (1–2%; F_(2,901)_ = 15. 6; η^2^ = 0.03), and arm reach (1–2%; F_(2,901)_ = 8.8; η^2^ = 0.02). The U14 female basketball players were also characterized by lower (*p* < 0.001, minimum effect) age at PHV (by 0.25/yrs) compared to U15 players.

**Table 1:** Descriptive statistics of female basketball players by chronological age and results of ANOVA analysis comparing age groups

Variables		1. U13 (n = 267)	2. U14 (n = 370)	3. U15 (n = 267)	F (*p*)	η^2^	d
Chronological age (years)	M	13.06	13.95	14.82	–	–	–
	S.E	0.02	0.02	0.02			
APHV (years)	M	11.65	11.78	12.03	72.1 (***)	0.14	1v2v3
	S.E	0.02	0.02	0.02			
Body height (cm)	M	165.3	167.7	167.8	15.6 (***)	0.03	1v2,3
	S.E	0.4	0.3	0.4			
Body mass (kg)	M	55.0	58.2	59.6	21.2 (***)	0.05	1v2,3
	S.E	0.5	0.4	0.5			
Standing reach (cm)	M	220.6	223.5	223.3	8.8 (***)	0.02	1v2,3
	S.E	0.6	0.5	0.6			
5 m (s)	M	1.243	1.221	1.205	4.6 (*)	0.01	1v3
	S.E	0.011	0.007	0.005			
10 m (s)	M	2.021	2.028	2.048	2.2	–	–
	S.E	0.012	0.008	0.007			
20 m (s)	M	3.585	3.551	3.539	5.8 (**)	0.01	1v3
	S.E	0.01	0.008	0.011			
Agility—S_1_ (s)	M	1.653	1.671	1.677	0.65	–	–
	S.E	0.015	0.013	0.015			
Agility—S_2_ (s)	M	5.768	5.736	5.672	2.2	–	–
	S.E	0.033	0.027	0.034			
Agility—S_3_ (s)	M	7.677	7.601	7.499	6.3 (**)	0.01	1v3
	S.E	0.035	0.03	0.036			
Agility—S_4_ (s)	M	10.082	9.953	9.754	13.6 (***)	0.04	1,2v3
	S.E	0.045	0.04	0.043			
Agility—S_5_ (s)	M	13.678	13.471	13.272	9.3 (***)	0.02	1v3
	S.E	0.063	0.059	0.064			
Agility—Total (s)	M	15.726	15.538	15.314	9.4 (***)	0.02	1,2v3
	S.E	0.068	0.059	0.062			
SVJ_max_ (cm)	M	255.3	259.8	260.0	17.7 (***)	0.04	1v2,3
	S.E	0.7	0.6	0.6			
VJ_max_ (cm)	M	263.7	268.1	268.7	15.6 (***)	0.04	1v2,3
	S.E	0.7	0.6	0.7			
SVJ (cm)	M	34.9	36.2	36.7	7.07 (**)	0.02	1v2,3
	S.E	0.3	0.3	0.4			
VJ (cm)	M	43.1	44.5	45.3	5.69 (**)	0.01	1v3
	S.E	0.5	0.4	0.5			
Yo-Yo Distance (m)	M	736	826	852	8.8 (***)	0.02	1v2,3
	S.E	20	18	22			
Yo-Yo VO_2max_ (ml/kg/min)	M	42.6	43.3	43.4	8.3 (***)	0.02	1v2,3
	S.E	0.2	0.1	0.2			

M mean; S.E. standard errors; SVJ standing vertical jump; VJ vertical jump; d significant differences between groups

^*^p < 0.05, **p < 0.01, ***p < 0.001

Furthermore, in the agility test, the U13 female players were slower (*p* < 0.001, minimum effect) than those from U15 group in S_4_ (by 3.4%; F_(2,901)_ = 13.6; η^2^ = 0.04). The other significant differences between sprint and agility values of this two groups were without effect size. Analysis revealed that the U13 female basketball players were slower compared to U15 for 5 m (by 3.1%; *p* < 0.05; F_(2,901)_ = 4.6; η^2^ = 0.01), 20 m (by 1.3%; *p* < 0.01; F_(2,901)_ = 5.8; η^2^ = 0.01), S_3_ (by 2.4%; *p* < 0.01; F_(2,901)_ = 6.3; η^2^ = 0.01), S_5_ (by 3.1%; *p* < 0.001; F_(2,901)_ = 9.3; η^2^ = 0.02) and considering the total agility test completion time (by 2.7%; *p* < 0.001; F_(2,901)_ = 9.4; η^2^ = 0.02). The U14 group was slower than U15 in S_4_ (by 1.4%, minimum effect) and total agility test completion time (by 1.5%, no effect).

Analysis of the results in the context of the jumping tests showed significantly lower values (1–5%) in SVJ_max,_, VJ_max_ (*p* < 0.001; minimum effect) and SVJ, VJ (*p* < 0.01; no effect) obtained by U13 compared to U15 players. An identical relationship (lower values within 1–4%) was observed in U13 compared to U14 in SVJ_max_, VJ_max_, and SVJ.

Compared to U14 and U15, female basketball players from the U13 group were also characterized by significantly (*p* < 0.001, but no effect) lower values of the distance covered (10–14%, F_(2,901)_ = 8.8; η^2^ = 0.02) and VO_2_max (1–2%, F_(2,901)_ = 8.3; η^2^ = 0.02) in the physical capacity test.

Table 2 shows the age-adjusted characteristics of female basketball players in relation to years after PHV (Table 2). In the group of U13 basketball players who were in PHV2, significantly (*p* < 0.001, minimum effect) higher values of body height (by 6.5%; F_(1,263)_ = 28.7; η^2^ = 0.18), arm reach (by 6.4%; F_(1,263)_ = 18.2; η^2^ = 0.12) and SVJmax (by 5.4%; F_(1,263)_ = 8.1; η^2^ = 0.06) were found compared to PHV1. In the same comparison there were also significantly (*p* < 0.05, but no effect) higher values of VJmax (by 4.7%; *p* < 0.05; F_(1,263)_ = 3.7; η^2^ = 0.03). In contrast, the PHV2 group performed worse in the 5 m sprint test (by 2.3%; *p* < 0.01; F_(1,263)_ = 5.5; η^2^ = 0.04; minimum effect) and endurance test, both in terms of the distance covered and VO_2_max (2–13%; *p* < 0.05; F_(1,263)_ = 3.3; η^2^ = 0.02; no effect).

**Table 2:** Age-adjusted means (standard errors in parentheses) by maturity timing within age groups and results of ANCOVAs with decimal age as the covariate

Variables	U13		F (*p*)	η^2^	U14			F (*p*)	η^2^	d	U15		F (*p*)	η^2^
	PHV1 (n = 154)	PHV2 (n = 113)			PHV1 (n = 22)	PHV2 (n = 264)	PHV3 (n = 84)				PHV2 (n = 63)	PHV3 (n = 204)		
Body height (cm)	160.7 (0.3)	171.2 (0.4)	28.7 (***)	0.18	154.4 (1.3)	166.2 (0.2)	176.6 (0.7)	27.2 (***)	0.18	1v2v3	160.5 (0.6)	170.5 (0.3)	30.5 (***)	0.19
Body mass (kg)	51.6 (0.6)	59.8 (0.8)	2.2	–	48.8 (2.5)	57.0 (0.5)	65.7 (1.3)	3.6 (*)	0.03	1v2v3	54.4 (1.2)	61.4 (0.6)	2.1	–
Standing reach (cm)	214.8 (0.6)	228.6 (0.8)	18.2 (***)	0.12	204.1 (2.3)	221.7 (0.4)	235.1 (1.2)	17.5 (***)	0.13	1v2v3	213.5 (1.1)	227.1 (0.5)	20.3 (***)	0.13
5 m (s)	1.228 (0.016)	1.256 (0.020)	5.5 (**)	0.04	1.217 (0.047)	1.215 (0.009)	1.214 (0.024)	0.7	–	–	1.198 (0.012)	1.205 (0.006)	0.8	–
10 m (s)	2.030 (0.017)	2.018 (0.021)	2.3	–	2.014 (0.050)	2.028 (0.009)	2.033 (0.026)	0.1	–	–	2.043 (0.016)	2.047 (0.008)	1.0	–
20 m (s)	3.571 (0.015)	3.611 (0.018)	1.2	–	3.542 (0.052)	3.546 (0.010)	3.556 (0.026)	1.0	–	–	3.515 (0.025)	3.543 (0.013)	0.2	–
Agility—S_1_ (s)	1.643 (0.022)	1.659 (0.027)	0.2	–	1.776 (0.083)	1.672 (0.016)	1.648 (0.042)	4.3 (**)	0.03	1v2,3	1.664 (0.037)	1.678 (0.019)	0.8	–
Agility—S_2_ (s)	5.718 (0.047)	5.805 (0.059)	0.7	–	5.968 (0.172)	5.711 (0.032)	5.771 (0.088)	0.1	–	–	5.774 (0.081)	5.682 (0.041)	3.5 (*)	0.03
Agility—S_3_ (s)	7.617 (0.049)	7.711 (0.062)	2.5	–	7.722 (0.193)	7.598 (0.036)	7.604 (0.099)	0.5	–	–	7.515 (0.086)	7.534 (0.043)	2.3	–
Agility—S_4_ (s)	10.014 (0.063)	10.142 (0.079)	2.5	–	10.122 (0.252)	9.945 (0.047)	9.964 (0.129)	0.6	–	–	9.851 (0.102)	9.781 (0.051)	3.5 (*)	0.03
Agility—S_5_ (s)	13.583 (0.090)	13.769 (0.113)	1.8	–	13.822 (0.377)	13.450 (0.071)	13.475 (0.192)	1.1	–	–	13.389 (0.153)	13.304 (0.077)	2.1	–
Agility—Total (s)	15.625 (0.097)	15.844 (0.121)	2.2	–	15.815 (0.376)	15.518 (0.071)	15.586 (0.192)	0.7	–	–	15.392 (0.148)	15.351 (0.075)	1.8	–
SVJ_max_ (cm)	249.7 (0.8)	263.1 (1.0)	8.1 (***)	0.06	240.9 (2.9)	258.1 (0.5)	271.3 (1.5)	7.1 (***)	0.06	1v2v3	251.1 (1.3)	263.5 (0.6)	13.6 (***)	0.09
VJ_max_ (cm)	258.4 (0.9)	270.6 (1.1)	3.7 (*)	0.03	248.8 (3.2)	266.4 (0.6)	279.1 (1.6)	4.2 (**)	0.03	1v2v3	257.8 (1.5)	272.5 (0.7)	10.3 (***)	0.07
SVJ (cm)	34.9 (0.5)	35.1 (0.6)	0.8	–	36.7 (2.0)	36.4 (0.4)	36.2 (1.0)	1.3	–	–	37.6 (0.9)	36.5 (0.4)	0.1	–
VJ (cm)	43.6 (0.7)	42.0 (0.9)	2.3	–	44.6 (2.5)	44.8 (0.5)	43.9 (1.3)	1.7	–	–	44.3 (1.1)	45.5 (0.6)	0.4	–
Yo-Yo Distance (m)	780 (29)	682 (36)	3.3 (*)	0.02	916 (109)	833 (20)	730 (56)	6.7 (***)	0.05	1v2v3	867 (51)	833 (26)	0.9	–
Yo-Yo VO_2max_ (ml/kg/min)	43.0 (0.2)	42.1 (0.3)	3.3 (*)	0.02	44.1 (0.9)	43.4 (0.2)	42.5 (0.5)	6.7 (***)	0.05	1v2v3	43.4 (0.4)	43.3 (0.2)	1.0	–

SVJ standing vertical jump; VJ vertical jump; d significant differences between groups

^*^p < 0.05, ** *p* < 0.01, *** *p* < 0.001

Furthermore, compared to groups PHV1 and PHV2, U14 female basketball players in PHV3 had higher (*p* < 0.001, minimum effect) body height (by 14.4% and 6.3%, respectively; F_(2,364)_ = 27.2; η^2^ = 0.18), arm range (by 15.2% and 6%, respectively; F_(1,263)_ = 17.5; η^2^ = 0.13), SVJ_max_ (by 12.6% and 5.1% respectively; F_(2,364)_ = 7.1; η^2^ = 0.06). In the same comparison there were also significantly (but no effect) higher values of body mass (by 34.6% and 8.7%, respectively; *p* < 0.05; F_(2,364)_ = 3.6; η^2^ = 0.03) and VJ_max_ (by 12.6% and 4.8%, respectively; *p* < 0.01; F_(2,364)_ = 4.2; η^2^ = 0.03). An identical trend was recorded in the same variables in favor of PHV2 compared to PHV1, ranging from 7 to 17%. Compared to PHV1 and PHV2, significantly lower values (*p* < 0.001; F_(2,364)_ = 6.7; η^2^ = 0.05; minimum effect) of distance covered (by 20.3% and 12.4%, respectively) and VO_2_max (by 3.6% and 2.1%, respectively) in endurance test were observed in the PHV3 group. Also in these two parameters, favorable results were obtained by the PHV1 group compared to PHV2 (1–10%).

Compared to PHV2, the U15 basketball players from group PHV3 showed higher (*p* < 0.001, minimum effect) values of body height (by 6.2%; F_(1,263)_ = 30.5; η^2^ = 0.19), arm reach (by 6.4%; F_(1,263)_ = 20.3; η^2^ = 0.13), SVJ_max_ (by 4.9%; F_(1,263)_ = 13.6; η^2^ = 0.09), VJ_max_ (by 5.7%; F_(1,263)_ = 10.3; η^2^ = 0.07). More favorable results (p < 0.05, but no effect) were observed in two sections of the agility test S_2_, S_4_ (within 1–2%; F(_1,263)_ = 3.5; η^2^ = 0.03).

Comparison of age-, height-, and weight-adjusted means (ANCOVA) with reference to years after PHV (Table 3) showed favorable results in 20 m sprint test and higher results in all jumping ability and endurance tests (1–6%) in the group of U13 female basketball players in PHV1. An identical trend of higher values (minimum effect) was observed in U14 female players in PHV1 compared to PHV2 and PHV3 in arm reach (1–2%; *p* < 0.05), SVJ_max_ (7–9%; *p* < 0.01), VJ_max_ (1–3%, *p* < 0.01) and a more favorable result in the 20 m sprint test (2–5%; *p* < 0.01). In the same comparison there was also significantly (by 42–53%; *p* < 0.05, but no effect) higher value of SVJ. The opposite trend (p < 0.01, minimum effect) was observed for distance covered during the endurance test (by 17–19%), VO2_max_ (by 12–13%; *p* < 0.01) and 5 m sprint (by 3–10%; *p* < 0.05; but no effect). Compared to PHV3, U15 basketball players from the PHV2 group were characterized by lower values of all variables of jumping abilities (1–10%), endurance (3–15%), and weaker time in 10 m and 20 m speed tests (1–3%), and on the S4 section and in the entire agility test (2–4%).

**Table 3:** Age-adjusted means (standard errors in parentheses) by PHV within age groups and results of ANCOVAs with decimal age, hight and body mass as the covariate

Variables	U13		F (*p*)	η^2^	U14			F (*p*)	η^2^	d	U15			F (*p*)	η^2^
	PHV1 (n = 154)	PHV2 (n = 113)			PHV1 (n = 22)	PHV2 (n = 264)	PHV3 (n = 84)				PHV2 (n = 63)		PHV3 (n = 204)		
Standing reach (cm)	221.1 (0.7)	221.3 (0.8)	1.0	–	225.4 (5.6)	221.4 (0.3)	221.2 (1.8)	2.7 (*)	0.05	1v2,3		218.1 (1.3)	219.9 (0.5)	1.7	–
5 m (s)	1.244 (0.025)	1.258 (0.031)	0.2	–	1.326 (0.177)	1.216 (0.009)	1.284 (0.056)	1.8 (*)	0.03	1v2,3		1.216 (0.023)	1.202 (0.009)	1.3	–
10 m (s)	2.028 (0.027)	2.023 (0.033)	1.7	–	1.805 (0.190)	2.029 (0.009)	2.082 (0.060)	0.8	–	–		2.083 (0.030)	2.039 (0.012)	2.8 (**)	0.04
20 m (s)	3.579 (0.022)	3.619 (0.028)	2.9 (**)	0.06	3.473 (0.192)	3.548 (0.009)	3.624 (0.061)	2.9 (**)	0.06	1v2v3		3.539 (0.048)	3.513 (0.019)	3.2 (**)	0.05
Agility—S_1_ (s)	1.674 (0.034)	1.674 (0.043)	0.3	–	1.704 (0.318)	1.673 (0.016)	1.635 (0.101)	1.6	–	–		1.678 (0.072)	1.665 (0.028)	1.0	–
Agility—S_2_ (s)	5.716 (0.074)	5.771 (0.092)	0.4	–	5.341 (0.657)	5.712 (0.032)	5.858 (0.210)	0.6	–	–		5.788 (0.157)	5.641 (0.061)	1.7	–
Agility—S_3_ (s)	7.653 (0.077)	7.617 (0.097)	1.1	–	7.005 (0.739)	7.598 (0.037)	7.650 (0.236)	0.5	–	–		7.745 (0.165)	7.479 (0.064)	1.6	–
Agility—S_4_ (s)	10.063 (0.099)	10.049 (0.124)	1.1	–	9.630 (0.962)	9.946 (0.047)	10.096 (0.307)	0.6	–	–		10.067 (0.196)	9.734 (0.077)	2.5 (*)	0.03
Agility—S_5_ (s)	13.607 (0.141)	13.614 (0.176)	1.0	–	12.344 (1.436)	13.455 (0.071)	13.563 (0.458)	0.9	–	–		13.676 (0.294)	13.276 (0.115)	1.7	–
Agility—Total (s)	15.646 (0.152)	15.676 (0.190)	1.1	–	14.725 (1.435)	15.522 (0.071)	15.612 (0.458)	0.8	–	–		15.670 (0.285)	15.265 (0.112)	2.1 (*)	0.03
SVJ_max_ (cm)	256.4 (1.0)	255.0 (1.2)	2.0 (*)	0.05	277.2 (8.5)	257.8 (0.4)	255.2 (2.7)	4.9 (**)	0.09	1v2,3		254.8 (2.1)	257.6 (0.8)	3.3 (*)	0.03
VJ_max_ (cm)	264.8 (1.1)	262.6 (1.4)	6.1 (**)	0.06	270.4 (9.9)	266.1 (0.5)	264.2 (3.2)	4.8 (**)	0.09	1v2,3		260.2 (2.5)	266.2 (1.0)	3.4 (**)	0.03
SVJ (cm)	35.3 (0.7)	34.7 (0.9)	2.6 (*)	0.05	51.8 (7.4)	36.3 (0.4)	34.0 (2.4)	3.3 (*)	0.03	1v2,3		36.7 (1.6)	37.7 (0.6)	3.4 (**)	0.03
VJ (cm)	43.6 (1.0)	41.3 (1.3)	4.6 (**)	0.09	45.0 (9.3)	44.7 (0.5)	43.0 (3.0)	3.1 (*)	0.02	1v3		42.1 (2.2)	46.3 (0.8)	3.1 (**)	0.03
Yo-Yo Distance (m)	783 (43)	742 (54)	3.6 (**)	0.05	686 (117)	830 (20)	844 (127)	5.6 (**)	0.04	1v2,3		765 (97)	900 (38)	3.5 (**)	0.04
Yo-Yo VO_2max_ (ml/kg/min)	43.0 (0.4)	42.6 (0.5)	3.5 (**)	0.05	38.0 (3.3)	43.4 (0.2)	43.5 (1.0)	5.6 (**)	0.04	1v2,3		42.2 (0.8)	43.7 (0.3)	3.6 (**)	0.05

SVJ standing vertical jump; VJ vertical jump; d significant differences between groups

^*^ *p* < 0.05, ** *p* < 0.01, *** *p* < 0.001

The results of the backward stepwise multiple regression analysis are presented in Table 4. The presented model explained 2–10% of the variance in individual strength and conditioning tests (adjusted *R*^*2*^ = 0.02–0.10; *p* < 0.001). The time from PHV was a significant predictor (positive value of the normalized β coefficient) only for the 10 m test. Furthermore, age was a significant predictor for the 5 m speed test, 20 m speed test, agility test (negative β coefficient), and SVJ, VJ, and VO_2_max (positive β coefficient) tests. The predictor of interactions of body height and body mass was significant only for the SVJ test (negative coefficient β). Body mass alone was a significant predictor in the 10 m and 20 m tests (positive direction) and VJ, VO_2_max (negative direction). It was also found that body height was a significant predictor only in the 10 m speed test.

**Table 4:** Summary of the backward regression for motor skills and anthropometric variables by the female basketball players aged 13–15 years

Attempt	Predictor	Standardized β	Adjusted R^2^	F (p)
5 m	Chronological age	− 0.111	0.02	11.2 (*)
10 m	Body height	− 0.161	0.02	7.2 (*)
	Body mass	0.154		
	APHV	0.100		
20 m	Chronological age	− 0.156	0.06	27.1 (*)
	Body mass	0.214		
Agility_Total_	Chronological age	− 0.160	0.03	23.7 (*)
SVJ	Chronological age	0.161	0.04	17.2 (*)
	Mass*height interaction	− 0.143		
VJ	Chronological age	0.170	0.06	26.5 (*)
	Body mass	− 0.201		
Yo-Yo VO_2max_	Chronological age	0.201	0.10	42.8 (*)
	Body mass	− 0.261		

SVJ standing vertical jump; VJ vertical jump

^*^ *p* < 0.001

## Discussion

The aims of this study were (1) to identify the physical fitness and basic anthropometric characteristics of Polish female basketball players aged 13 to 15 years, (2) to show the effect of maturity timing on the performance in motor tests and basic body composition parameters, (3) to identify the index that contributes most to the prediction of performance in the tests of sprint, jumping ability, agility, and endurance.

The first aim of the study was to identify motor potential and basic anthropometric characteristics of the population of young female basketball players aged 13–15 years. Determination of these parameters is a starting point in the search for candidates for the participant of national teams or defining the characteristics needed or conducive to high performance. Talent identification programs are an integral part of the selection process for elite-level athletes and every sport has its set of variables being an important part of success [50]. Many sports clubs have their individual systems of selection according to the most important features in a given discipline or event. This rationale underlies the emergence of various recruitment and selection programs aimed at seeking, identifying, and developing talented individuals [51, 52]. Polish female basketball players from the U15 group achieved slightly better (compared to peers, the best basketball female players in Europe, participating in the youth championships of European Division A), or identical results (compared to female basketball players participating in the youth championships of European Division B) in 20 m sprint test [53]. Further, Polish female athletes were characterized by lower values of body height and body mass. On the other hand, a comparison of the values after correcting with body height revealed no significant differences [53]. Such a trend may indicate that the Polish players are more similar to their peers from Division B (2nd European League) teams than to Division A (1st European League). The basketball players from the U13 group also achieved better results (even considering age-adjusted values) in the 20 m sprint compared to their peers from Portugal [7].

The second study aim was to show the impact of maturity timing on the results of motor tests and basic body composition. Somatic built and physical fitness potencial determines, on the one hand, factors necessary to qualify an athlete for a given sport and, on the other hand, having optimal parameters for success in the sport. Analysis of values related to age-adjusted characteristics showed that in the PHV2 under 13-year-old group observed significantly higher SVJ_max_, VJ_max_ values but worse sprint (5 m) and endurance results (distance covered, VO_2max_) than PHV1 group. U14 female basketball players in PHV3 had significantly higher SVJ_max_, VJ_max_ results compared to groups PHV1 and PHV2 and lower values of endurance test (distance covered and VO2_max_). Limited study suggest that less mature girls perform better than more maturing girls in some tasks, but overall maturity—associated variation is not consistent across tasks and ages [54]. The U15 basketball players from PHV3 compared to PHV 2 group showed higher values of SVJ_max_, VJ_max_ and agility test (S_2_, S_4_). This is consistent with previous researche where more mature players are typically characterized by higher performance in speed, agility, and lower limb power [7, 55].

Similar trend was shown in ANCOVA results (age, body height, and body mass as covariates) where in the U13 female basketball players observed significantly higher sprinting (20 m), jumping ability and endurance tests results of the PHV1 group. Better results was observed in U14 female players in PHV1 compared to PHV2 and PHV3 in 20 m and jumping tests but opposite trend was observed for 5 m sprint and endurance test (distance covered and VO2_max_). U15 basketball players from the PHV3 group were characterized by better results of jumping abilities, endurance, 10 m and 20 m sprint and agility (total, S_4_) tests. In this context, it is important to take biological development into account, since differences resulting from this development are most often overlooked and thus early maturers are promoted. Selection carried out in this way is often flawed if decisions are made before the maturation process is complete [56].

The third aim of the study, which was to identify the indices most useful in predicting the level of motor preparation, allowed for the conclusion that chronological age, height, weight, weight-height interaction, and the time from PHV accounted for 2–10% of the variance in individual physical fitness tests. Maturity timing was a significant predictor only in sprint test (10 m) but body height and body mass were the most significant predictors in this test. Rest results showed that chronological age (5 m, 20 m, agility, SVJ, VJ, and VO_2max_ tests), body height (10 m), body mass (10 m, 20 m, VJ, VO_2max_), and the interaction between body mass and height (SVJ) were significant (adjusted *R*^*2*^ = 0.02–0.10; *p* < 0.001) predictors of motor skills. In general, considering the adjusted R^2^ value, most of the differences in the results obtained by female basketball players were not explained by these variables. There is extensive literature on the analysis of selected motor and functional fitness characteristics in correlation with the level of physical development in adolescent girls, however mainly in general non-athlete populations [57, 58]. In contrast, the results of our study are consistent with previous findings and show that much of the variation in sport-specific functional abilities and skills is not explained by age, puberty, and body size [8, 10] and for young training girls, understanding the complex interactions of physical development, especially body mass and height, with sport-specific motor abilities and biological age is a key component of sporting success [7].

The objectivity of inferring developmental capabilities depends largely on a comprehensive assessment of the status of various functional systems involved in a specialized task. Such an assessment of physical and mental preparation, in addition to the generally accepted indicators, should take into account chronological, biological, and sport age (accumulated training and competitive experience in sport) [19], individual rate of performance development, indicators of physical development and motor activity at the stage before the examination and the assessment of their prospective capabilities [59]. Particular caution should be exercised in the selection based on index values obtained before puberty. Most of the changes occurring during puberty (e.g. aerobic and anaerobic capacity, fitness, body composition) are non-linear [60]. Results showed that only a third of international pre-junior athletes reappeared as senior athletes, confirming the difficulties of predicting late success based on early identification and selection [61].

Further, both somatic, motor and developmental parameters should not be treated as an absolute selection criterion, especially in sports where the results are an effect of multidimensional dependencies of various variables [62]. The superior performance here may result from individually varying relationships between innate determinants of athletic performance and environmental factors [59].

The most important limitation of the present study was not including the menarche age. However, we know from other studies that menarche is a late adolescent event that occurs, on average maturers, about a year or so after PHV [63, 64]. This regularity was also confirmed by longitudinal studies of Polish girls (n = 176, Polish ancestry, Poznań growth study) where the mean time interval between PHV and menarche was 1.28 years [64]. At Kaczmarek’s study age at menarche was only weakly correlated with height at critical points of the adolescent spurt and reversely correlated with velocity – the earlier the menarche the higher the height velocity [64]. In other longitudinal studies of trained girls (n = 23) and their untrained peers (n = 26; Polish ancestry, Warsaw growth study) interval between PHV and menarche was 1.2 ± 0.6 and 1.1 ± 0.5, respectively [63]. Peak height velocity and menarche occur, on average maturers, slightly later in trained girls (APHV—12.0 ± 0.8 active; 11.8 ± 0.7 non active), but the differences were not significant [63]. In another study of USA population (n = 156, 80.6%—white race and 19.4%—nonwhite race) the mean age at PHV was 12.1 ± 1.4 years for females and the majority of girls (69.1%) had achieved PHV by Tanner stage 3 [65].

Another limitation of present study was that we did not account for factors that may influence linear growth and pubertal timing such as obesity, which can accelerate puberty in girls [66]. In our study did not also consider analysis depending on sport age (accumulated training and competitive experience in sport) [19]. Finally, all equations used to predict APHV (maturity shift) or APHV have the same major limitations [67, 68]. The advanced maturity timing and the relatively narrow range of variation in predicted age at peak height velocity may undermine its utility and effectiveness in talent identification and development programs when applied at a specific time point [6, 15].

Given the above, it seems that knowledge of APHV of players is important to identify differences in motor potential caused by developmental changes [69]. However, it must be complemented by the use of methods related to the evaluation of technique within individual specializations [70]. Another concern to consider is the using of methods to assess the effect of body size (tridimensional charactersitics of players) on performance fitness test results, such as allometric scaling. In future studies of this type, especially considering adolescent girls' groups, an attempt should be made to combine factors determining performance, such as hormonal status, body components, perceptual-cognitive elements, tactical skills, and sport age. Study the relation of APHV and performance using Bayesian methods should be provided to interpretation about trends of outcomes and controlling the influences of multilevel clustering [19]. Finally, longitudinal studies of a group of female basketball players are desirable in order to describe their career progression (whether they reach or not professional level, drop-out from discipline and why), in or between seasons comparison, assess some psychological dimensions (to understand what relationships are the most important in the young players development).

## Conclusions

The results of this study improve our understanding how maturity timing influences on the performance in motor tests and basic body composition parameters in youth female basketball players. The status of reference to years after PHV has a particular effect on performance during jumping test, endurance test, and 20 m speed test in all three (U13-U15) age categories. The time from peak height velocity (PHV) was a significant predictor only in the 10 m speed test, but height and weight were the most significant. Chronological age (5 m, 20 m, agility, SVJ, VJ, and VO_2max_ tests), body height (10 m), body mass (10 m, 20 m, VJ, VO_2max_), and the interaction between body mass and height (SVJ) were significant predictors of motor skills. The results can help the coaches to personalize training programs and to adapt the training content to the biological age of the players.

## Data Availability

Full access to data on request (karol.gryko@awf.edu.pl).

## Abbreviations

APHV: Age at peak height velocity
PHV: Peak height velocity
SVJ: Standing vertical jump
VJ: Vertical jump
